# Catalytic Conversion of Carbohydrates into 5-Hydroxymethylfurfural by Phosphotungstic Acid Encapsulated in MIL-101 (Cr, Sn) Catalyst in Deep Eutectic Solvents

**DOI:** 10.3390/ijms241411480

**Published:** 2023-07-14

**Authors:** Wei Mao, Jiawen Hao, Lingyu Zeng, Hao Wang, Hao Xu, Jinghong Zhou

**Affiliations:** Guangxi Key Laboratory of Clean Pulp & Papermaking and Pollution Control, School of Light Industrial and Food Engineering, Guangxi University, Nanning 530004, China; maowei@st.gxu.edu.cn (W.M.);

**Keywords:** bimetal–organic frameworks, phosphotungstic acid, loading, cassava starch, catalysis

## Abstract

Herein, we report the synthesis of bimetal–organic frameworks (BMOFs) with both Brønsted and Lewis acidities, in which phosphotungstic acid (PTA) was encapsulated in BMOFs. It is efficient in converting starch to 5-hydroxymethyl-furfural (HMF) in deep eutectic solvents (DESs) such as choline chloride and formic acid. The highest yield of HMF (37.94%) was obtained using P_0.5_/BMOFs_1.0_ to catalyze starch in a mixed solvent system comprising DESs and ethyl acetate (EAC) (*v*/*v*; 2:3) at 180 °C and a reaction time of 10 min. Employing a DES as a cocatalyst and solvent reduced the use of organic solvents. The catalyst showed adequate reusability, and the HMF yield only decreased by 2.88% after six cycles of reuse compared with that of the initial catalyst. This study demonstrates the application potential of BMOFs in the conversion of biomass to useful molecules with commercial and/or research value.

## 1. Introduction

5-hydroxymethyl-furfural (HMF) is a significant platform compound with a furan ring, an aldehyde group, and a hydroxymethyl group in its molecular structure. HMF is chemically reactive and used in the preparation of various high-value-added chemicals through hydrogenation, oxidation, and condensation [1]. HMF can be readily produced from monosaccharides, namely, fructose, glucose, mannose, and galactose [2], in high yields [3]. However, the high cost of monosaccharides limits their application in the widespread industrial production of HMF. Fortunately, these monosaccharides can be derived from polysaccharides, such as starch, cellulose, and hemicellulose, and carbohydrates, such as wood and lignocellulose [4,5,6]. As this approach to producing monosaccharides is sustainable from the perspective of environmental preservation, it is widely used in the field of biomass refinement for the preparation of HMF. For the green applications of biomass resources, developing novel chemical methods for the conversion of glucose-based biomass to HMF at a low cost with high efficiency is crucial. Using the combination of Brønsted and Lewis acid catalysts, glucose is isomerized to fructose, which is easier to convert to HMF than glucose.

Numerous studies have been focused on determining innovative and efficient schemes for the preparation of HMF, including the use of catalytic materials, solvent systems, raw materials, and appropriate reaction conditions [7,8,9,10,11]. Under acidic conditions, starch is hydrolyzed into glucose, which is first isomerized into fructose by a Lewis acid and then dehydrated into HMF by a Brønsted acid [12,13]. However, incorporating Brønsted and Lewis acidities into a catalyst for carbohydrate conversion, although useful, is very challenging. Li et al. [14] used a silica–aluminum composite catalyst doped with Nb to convert glucose and found that weak Lewis acid could promote the formation of HMF. When the ratio of Brønsted acid to Lewis acid was 0.68, the HMF selectivity could reach 71% at 92.6% conversion of glucose. Zhao et al. [15] used insoluble heteropolysate Cs_2.5_H_0.5_PW_12_O_40_ as the chemical agent and fructose as the reaction substrate. At the reaction temperature of 115 °C, the yield of HMF could reach 77.5%, and they also used Ag_3_PW_12_O_40_ to catalyze glucose to prepare HMF. At the reaction temperature of 130 °C, the yield of HMF was 76.3%. Rahaman, Mohammad Shahinur et al. [16] used a phosphotungstic acid-encapsulated MIL-101(Al) -NH_2_ catalyst to bring Brønsted acid sites close to Lewis acid sites. The encapsulated catalysts had a high HMF selectivity of 58% at 44% glucose conversion at 120 °C in [C_4_C_1_im]Cl. Phosphotungstic acid (PTA) is a Keggin-type heteropoly acid with excellent Brønsted acidity and adequate thermal stability in the solid state [17]. Although PTA exhibits adequate performance in acid-catalyzed reactions, its separation from products is difficult (similar to most heterogenous catalysts), which affects its recycling performance. To address this separation problem, loading PTA onto suitable supports is an effective approach. Therefore, the study of PTA-supported catalysts is promising in the acid-catalytic research field. Metal–organic frameworks (MOFs), a new type of porous material, have attracted considerable attention as heterogeneous catalysts, owing to their porosity, high specific surface area, adjustable pore size, and structural diversity [18]. Owing to the presence of large amounts of unsaturated metal centers, many MOFs have been used as Lewis acid catalysts [19,20,21]. Studies on MOFs with both Brønsted and Lewis acidities for glucose conversion are scarce [22,23]. MOFs based on chromium(III) terephthalate (MIL-101) have high thermal stability and a large mesoporous cage (2.9–3.5 nm) [24], which enables PTA (diameter of 1.4 nm) transport through microporous windows (1.2–1.6 nm) [25]. Therefore, MIL-101 has been considered an ideal material support for PTA. In a previous study, PTA was encapsulated in MIL-101(Cr) (PTA/MIL-101) and showed an adequate catalytic performance in the esterification of *n*-butanol with acetic acid [26]. Zhang et al. [27] synthesized PTA/MIL-101(Cr), used it as a solid acid catalyst in the dehydration of carbohydrates in ionic liquids, and reported an HMF yield of 79%. The application of Cr MOFs [28,29] in chemical products is limited by their poor water stability and the use of toxic inorganic reagents such as hydrofluoric acid [30] in their synthesis.

Solvent systems [4] have also been developed to dissolve substrates and products. Ionic liquids (ILs) and deep eutectic solvents (DESs) can act as alternative media for solubilization or catalysis during biomass processing [31,32]. Using ILs or DESs as solvent/cosolvent and/or catalyst/cocatalyst systems to convert carbohydrates into HMF can improve the yield and conversion of HMF and minimize the harmful effects of volatile solvents on the environment. Delbecq et al. [33] used a betaine/formic acid–methyl isobutyl ketone (MIBK)/H_2_O bidirectional system with microwave heating to catalyze the conversion of fructose, glucose, starch, and microcrystalline cellulose to HMF with yields of 82%, 55%, 54%, and 47%, respectively. In addition, choline chloride is inexpensive and readily available for the industrial-scale preparation of HMF from carbohydrates. DESs are a novel solvent system for the solubilization and catalytic conversion of various carbohydrates.

In the present study, P_Y_/BMOFs, a catalyst with Brønsted and Lewis acidities, was synthesized via the in situ loading of PTA as the acid active center. Brønsted and Lewis solid acids were investigated as catalysts in the conversion of starch to HMF, with choline chloride/formic acid (CC/FA) and ethyl acetate (EAC) as the solvent system. Several reaction parameters, including the reaction temperature, time, and catalyst dosage, were examined in the presence of P_Y_/BMOFs and DESs.

## 2. Results and Discussion

### 2.1. Characterization of Catalyst

**Figure 1:**
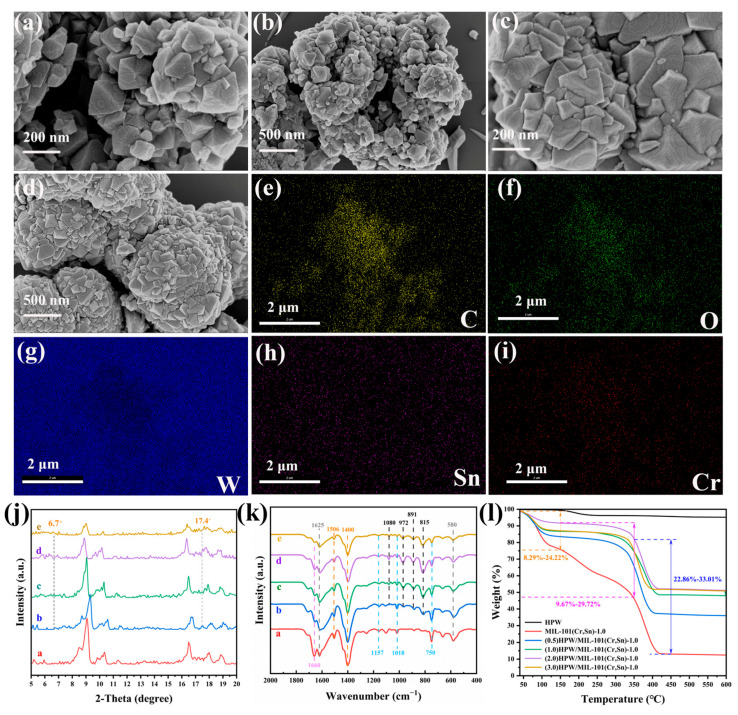
(**a**,**b**) SEM images of P_0.5_/MIL-101(Cr, Sn)_1.0_; (**c**,**d**) SEM images of P_2.0_/MIL-101(Cr, Sn)_1.0_; (**e**–**i**) EDS elemental maps of P_0.5_/MIL-101(Cr, Sn)_1.0_ for C, O, W, Sn, Cr Mapping diagram; (**j**) XRD patterns of the samples (a: MIL-101(Cr, Sn)_1.0_, b: P_0.5_/MIL-101(Cr, Sn)_1.0_, c: P_1.0_/MIL-101(Cr, Sn)_1.0_, d: P_2.0_/MIL-101(Cr, Sn)_1.0_, e: P_3.0_/MIL-101(Cr, Sn)_1.0_); (**k**) Infrared spectra of the samples (a: MIL-101(Cr, Sn)_1.0_, b: P_0.5_/MIL-101(Cr, Sn)_1.0_, c: P_1.0_/MIL-101(Cr, Sn)_1.0_, d: P_2.0_/MIL-101(Cr, Sn)_1.0_, e: P_3.0_/MIL-101(Cr, Sn)_1.0_); (**l**) Thermogravimetric curves of P_Y_/MIL-101(Cr, Sn)_1.0_ materials with different PTA loadings.

Phosphotungstic acid encapsulated in MIL-101 (Cr, Sn) catalyst (P_Y_/BM_1.0_) was characterized by XRD, FT-IR, XPS, SEM, BET, elemental analysis, and acid–base titration. The SEM images of P_0.5_/BM_1.0_ and P_2.0_/BM_1.0_ are shown in Figure 1a–d. Figure 1a shows that P_0.5_/BM_1.0_ has a three-dimensional octahedral structure and a smooth surface. The doping of phosphotungstic acid (PTA) has little effect on the material morphology. However, the bonding between P_Y_/BM_1.0_ particles is stronger than that between BM_1.0_ particles. According to EDS analysis of the sample (Figure 1e–i), P_0.5_/BM_1.0_ contains five elements: C, O, W, Sn, and Cr. These elements are widely distributed, but their quantities are not evenly distributed. It can be seen that the brightness of W is the highest, indicating that its content is the largest, mainly from the PTA. The content of C and O is very high, and the distribution is also wide, mainly from Cr(NO_3_)_3_·9H_2_O and *p*-phthalic acid. The distribution of Cr and Sn is also very uniform, but the relative content is small.

The main diffraction peak of P_Y_/BM_1.0_ (Figure 1j) was similar to that of BM_1.0_, suggesting that the PTA encapsulated into BM_1.0_ without disturbing the MOFs structure [26]. Furthermore, the intensity of the characteristic peak decreased with the increasing PTA loading. Figure 1k shows the FT-IR spectra of P_Y_/BM_1.0_ with different loadings of BM_1.0_ and PTA. It can be seen from Figure 1k that pure PTA has P–O, W–O_d_, W–O_b_–W, and W–O_c_–W stretching vibrations in the frequency bands of 1080 cm^−1^, 986 cm^−1^, 889 cm^−1^, and 804 cm^−1^_,_ respectively. The vibrations representing P–O, W–O_d_, W–O_b_–W, and W–O_c_–W of the samples exhibited differences from those of PTA. Compared with the bands of pure PTA, those ascribed to W–O_b_–W and W–O_c_–W shifted to higher wavenumbers and were more intense. The W–O_d_ bands of the samples were shifted to lower wavenumbers (972 cm^−1^) and were more intense, demonstrating that the P_Y_/BM_1.0_ catalyst achieved the immobilization of the PTA, and the PTA retained the Keggin anion after encapsulation [24,27].

The acid density and acid strength of the sample were determined by acid–base titration and initial potential (Table 1). The maximum initial potentials of PTA and BM_1.0_ were 588 mV and −45 mV, respectively. Compared with the initial potential of BM_1.0_, that of P_0.5_/BM_1.0_ decreased to 142 mV. As the PTA loading increased from 12.78% to 20.59%, the initial potential increased from 180 mV to 193 mV. Theoretically, the pore volume of the catalyst affects the PTA content. As can be seen in Table 2, the BET specific surface area and pore volume of BM_1.0_ in this study (*S*_BET_ = 2826 m^2^/g and *V*_total_ = 1.31 cm^3^/g) were similar to those of MIL-101 reported in the literature [34]. The diameter of the PTA was between 1.3 nm and 1.4 nm [35]. As the amount of PTA loading increased, the *S*_BET_ decreased from 2826 m^2^/g to 895 m^2^/g, *V*_total_ decreased from 1.31 cm^3^/g to 0.48 cm^3^/g, and the average pore size decreased from 4.02 nm to 2.58 nm. This trend was caused by the immobilization of the PTA within the channels of BM_1.0_, resulting in decreased *S*_BET_ and *V*_total_. It was observed that when the loading of the PTA increased from 12.78% to 20.59%, the decrease in *S*_BET_ and *V*_total_ was small (Table 2), which had the same trend as the initial potential.

Thermogravimetric analysis data show how the weight loss progresses with the different PTA loadings (Figure 1l). The samples have three stages of weight loss when the temperature rose from 30 °C to 450 °C. Catalysts lost weight at a rate of 8.29% to 24.22% when they went from room temperature to 150 °C due to the detachment of physiosorbed water molecules from BM_1.0_ and P_Y_/BM_1.0_. The second weight-loss phase occurred from 150 °C to 350 °C, with a mass loss between 9.67% and 29.72%. This represents the crystal water removal and the partial decomposition of the terephthalic acid ligand [36]. When the temperature went from 310 °C to 450 °C, the decomposition of ligands led to the collapse of the samples, resulting in a sharp reduction in the sample weight [34]. When the temperature exceeded 450 °C, the weight of the catalyst stabilized. The above data prove that the catalysts with different PTA loadings can still maintain good thermal stability.

**Figure 2:**
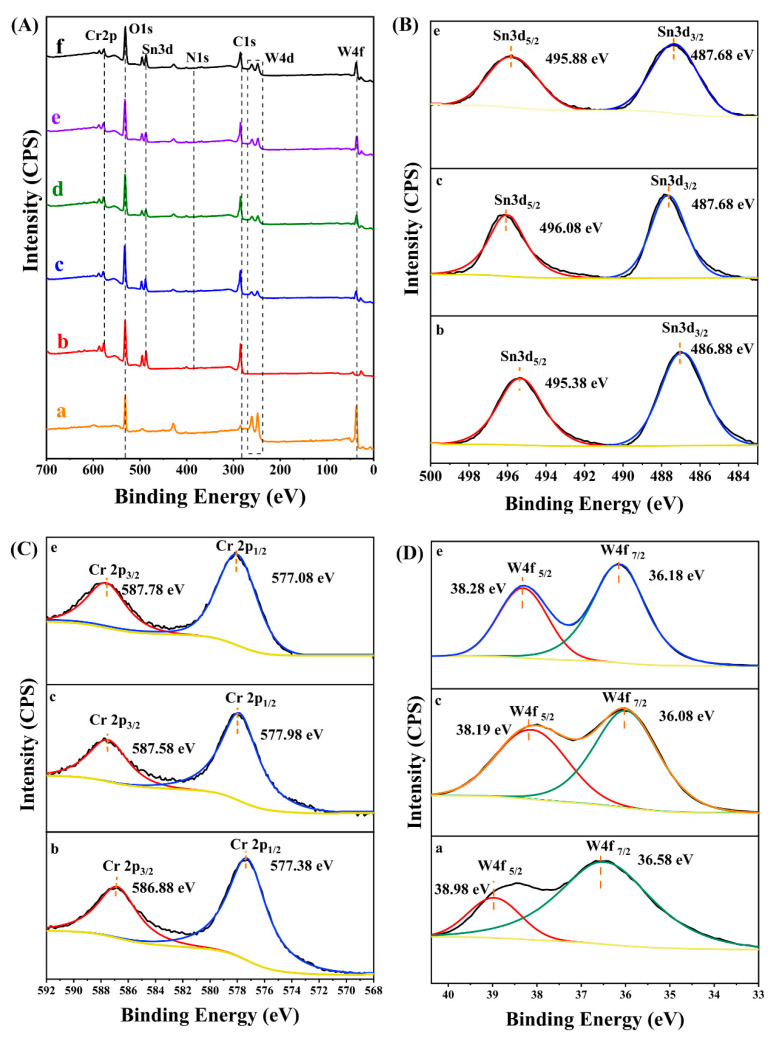
XPS energy spectra of the samples, (**A**). full spectrum; (**B**). W4f; (**C**). Sn3d; (**D**). Cr2p. (a: PTA, b: MIL-101(Cr, Sn)_1.0_, c: P_0.5_/MIL-101(Cr, Sn)_1.0_, d: P_1.0_/MIL-101(Cr, Sn)_1.0_, e: P_2.0_/MIL-101(Cr, Sn)_1.0_, f: P_3.0_/MIL-101(Cr, Sn)_1.0_).

The XPS spectra of PTA, BM_1.0_, and P_Y_/BM_1.0_ were measured to investigate the chemical bonding states of Cr 2p, O 1s, Sn 3d, N 1s, C 1s, and W 4f (Figure 2). The Sn 3d spectrum (Figure 2B) shows two characteristic peaks centered at 486.78 eV and 495.28 eV, which are attributable to Sn 3d_5/2_ and Sn 3d_3/2_, respectively, and are characteristic of tetravalent Sn(IV) [37]. Similarly, peaks located at 577 eV and 587 eV are attributable to Cr 2p_1/2_ and Cr 2p_3/2_, respectively (Figure 2C) [38]. The W 4f spectrum of pure PTA (Figure 2D) exhibited two main characteristic peaks at 38.98 eV and 36.58 eV, corresponding to W 4f_5/2_ and W 4f _7/2_, respectively. Compared with those of pure PTA, the intensities of the characteristic peaks of W 4f_5/2_ and W 4f_7/2_ in P_Y_/BM_1.0_ declined after the immobilization of PTA. This suggests that the loading of PTA on BM_1.0_ has not destroyed the original skeleton structure of MIL-101(Cr, Sn)_1.0_. In order to further confirm the elemental content, XPS was used to analyze the samples. The elemental analysis of the BM_1.0_ and P_Y_/BM_1.0_ samples is detailed in Table 1. The contents of P in the products of four kinds of MOFs with different PTA loadings were 0.45, 0.56, 1.04, and 0.90, and the contents of W were 1.55, 2.04, 3.28, and 3.03.

### 2.2. Effect of Solvents

**Figure 3:**
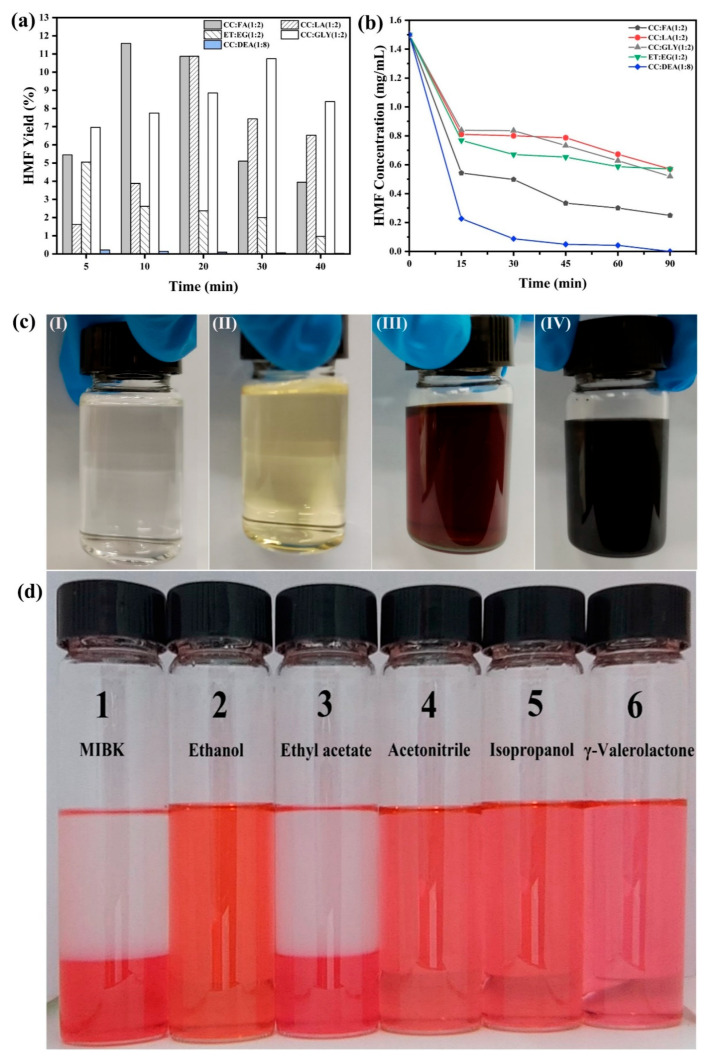
(**a**)Effect of different types of DESs on starch conversion to HMF; (**b**) Stability of HMF in DESs; (**c**) Color change in DES solution; (**d**) DESs and organic solvent compatibility (Add methyl orange reagent to DES, then mix with organic solvent, stir at 60 °C for a period of time, and let it stand at room temperature to observe whether it can be stratified).

The reaction solvent is a key parameter in the production of 5-hydroxymethyl-furfural (HMF) from polysaccharides. Recently, deep eutectic solvents (DESs) [32] have been used as a “green” solvent to replace the volatile organic solvents that are typically used for the conversion of starch to HMF. In previous studies [39,40], the conversion of starch into HMF was carried out in a series of DESs. The results are shown in Figure 3a. Among the five types of DES systems, choline chloride/formic acid (CC/FA) had the highest yield of HMF, achieving 11.58% after 10 min reaction time. The lowest yields were obtained with choline chloride/diethanolamine (CC/DEA). In addition, CC/FA systems exhibited the highest solubility for starch, compared with the four other DESs. However, HMF was unstable in the DES systems (Figure 3b), which was also reflected by the lack of color change in the DES solution without HMF after heating (Figure 3c). In contrast, the color of the DES solution with HMF changed from white to coffee to black after heating. If the generated HMF is not extracted from the solution in time, it is prone to side reactions, producing a large amount of humins [6,41]. In order to obtain a satisfactory HMF yield, the generated HMF is rapidly transferred to organic solvent by extraction to avoid the decomposition of HMF [42,43]. As shown in Table S2, ethanol, acetonitrile, isopropanol, and γ-valerolactone (GVL) were all miscible with DESs and difficult to separate. Methyl isobutyl ketone (MIBK) and ethyl acetate (EAC) could be separated from DES and organic solvents, and there was no DES residue in the evaporated organic solvent (Figure 3d), suggesting that MIBK, EAC, and DESs were immiscible and suitable as extractants for the catalytic reaction in DES. EAC and MIBK have a similar extraction ability for HMF, as well as low solubility and good stability in DESs. EAC has a lower boiling point (77 °C) than MIBK (117 °C) and was used as the next reaction solvent.

The effect of the volume ratio of DESs/EAC to starch on the yield of HMF and separation coefficient was studied at a P_0.5_/BM_1.0_ concentration of 0.225% (based on the mass of the solution) and temperature of 180 °C for 10 min, as shown in Table 3. When the volume ratio of CC/FA and EAC was 8/12, the yield of HMF reached a maximum value of 37.94% and the separation coefficient decreased to 37.46%. The separation coefficient increased with the increasing volume of DESs and decreasing volume of EAC extractant, suggesting that EAC played an extractive role in the reaction and promoted the reaction toward the production of HMF. However, for economic reasons, the optimal volume ratio of DESs and EAC was 8/12. Therefore, CC/FA not only acted as a solvent and cocatalyst in the catalytic process, but also reduced the use of the organic solvent.

### 2.3. Catalytic Performance

**Figure 4:**
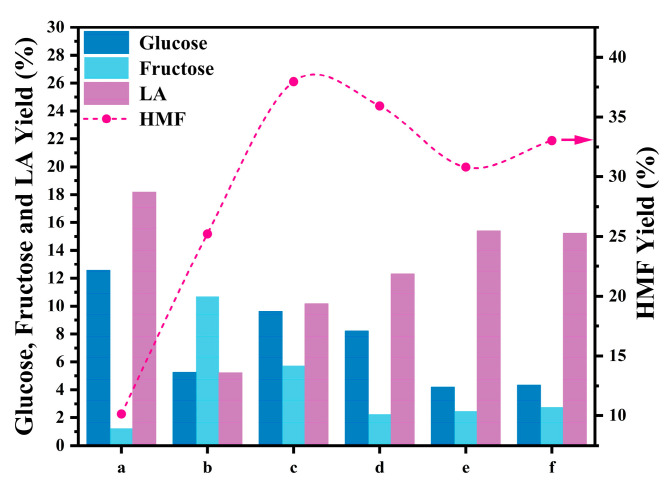
Effect of different PTA loadings on HMF, glucose, fructose, and LA yields. (a: PTA, b: BM_1.0_, c: P_0.5_/BM_1.0_, d: P_1.0_/BM_1.0_, e: P_2.0_/BM_1.0_, f: P_3.0_/BM_1.0_. Conditions: 0.15 g starch, catalyst concentration of 0.225%, 180 °C, 10 min, 8 mL CC/FA, 12 mL EAC).

The catalytic efficiencies of P_Y_/BM_1.0_ for HMF synthesis were studied at 180 °C for 10 min (Figure 4). Using the PTA catalyst, a 10.13% yield of HMF, 12.55% yield of glucose, and 18.17% levulinic acid (LA) were achieved. The formation of a small amount of fructose was detected during the experiment. The results suggest that in the presence of only a Brønsted acid, and no Lewis acid, the catalytic conversion efficiency of starch to glucose was higher, while that of glucose to HMF was lower. BM_1.0_ gave yields of 25.21% for HMF, 5.23% for glucose, and 10.70% for fructose. Brønsted acids are essential for the conversion of starch to glucose, and Lewis acids play an important role in the isomerization of glucose to fructose, as well as fructose conversion to HMF [44,45,46,47]. PTA is a typical Brønsted acid, while BM_1.0_ is a Lewis acid, and the introduction of PTA into BM_1.0_ changes the proportion of Brønsted and Lewis acids on the catalyst (Table 1). The maximum yield of HMF (37.95%) was obtained when the catalyst used was P_0.5_/BM_1.0_, which also showed excellent catalytic activity in glucose and fructose synthesis. HMF yields of 35.93%, 30.80%, and 33.12% were obtained when using P_1.0_/BM_1.0_, P_2.0_/BM_1.0_, and P_3.0_/BM_1.0_, respectively. As seen in Table 1, when the PTA loading increased from 9.71% to 20.59%, the Brønsted acidity of the catalyst gradually increased. However, the yields of HMF and glucose decreased with the increasing Brønsted acidity of the catalyst (Figure 4), while the yield of the by-product LA increased. One possible reason for this phenomenon is that acidic DES (CC/FA) was used as the reaction solvent in the experiment. Thus, side reactions, such as HMF reactions with water to synthesize LA and FA, are promoted by the increased Brønsted acidity of the catalyst. The HMF that is not extracted into EAC in time is partially decomposed into by-products such as humins and LA. Another reason is that the increase in the PTA loading leads to the decrease in the *S*_BET_ and pore size of the catalyst (Table 2), and a catalyst with large enough pores allows glucose and other products to freely diffuse [48]. This result suggests that bimetallic MOF catalysts with double acid sites in acidic solution is promising as a combined catalyst in a one-pot reaction producing HMF from starch.

**Figure 5:**
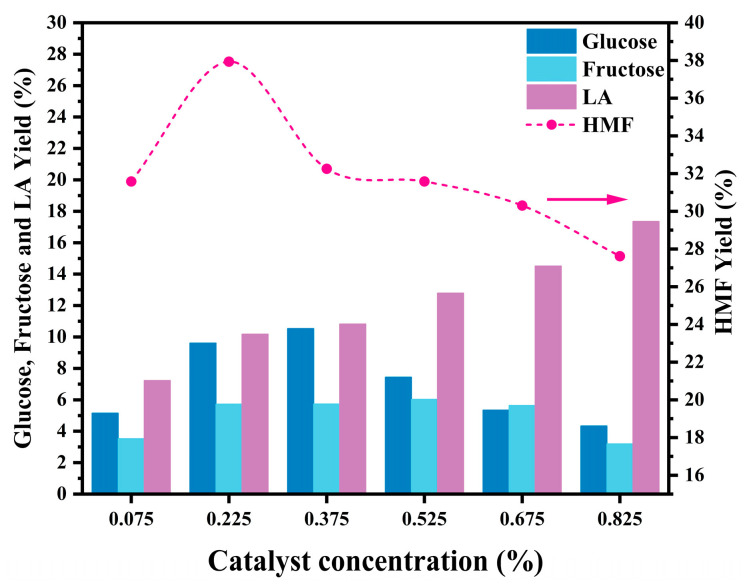
Effect of catalyst concentration on starch dehydration. (The catalyst concentration is calculated based on the mass of the solution, which is the percentage of the mass of the solvent. Reaction condition: catalyst: 0.15 g starch, P_0.5_/BM_1.0_, 180 °C, 10 min, 8 mL CC/FA, 12 mL EAC).

Because P_0.5_/BM_1.0_ shows the highest catalytic activity for starch hydrolysis into glucose, as well as glucose-to-fructose isomerization, we used it as the catalyst for the subsequent experiments. The effect of the catalyst dosage on the conversion of starch into HMF was studied, and the results are shown in Figure 5. It can be seen that the highest yield of HMF (37.94%) was achieved under a relatively low catalyst concentration (0.225%). A partial decrease from 37.94% to 27.61% in the HMF yield was detected after increasing the concentration of the catalyst from 0.225% to 0.825%. The change in trend was the same for the glucose and fructose yields, both of which increased initially, then decreased. There was a different trend in the LA yield, which showed a rapid increase with the increasing catalyst dosage. These results suggest that the decrease in the HMF yield at the higher P_0.5_/BM_1.0_ dosage was due to the by-products from the side reactions. This could be attributed to the presence of acidic DESs in the reaction solution, and the excess P_0.5_/BM_1.0_ provides more available acid sites, promoting the decomposition of HMF or the polymerization of HMF and glucose [6,49].

### 2.4. Effects of Reaction Time and Temperature on the Yield of HMF

**Figure 6:**
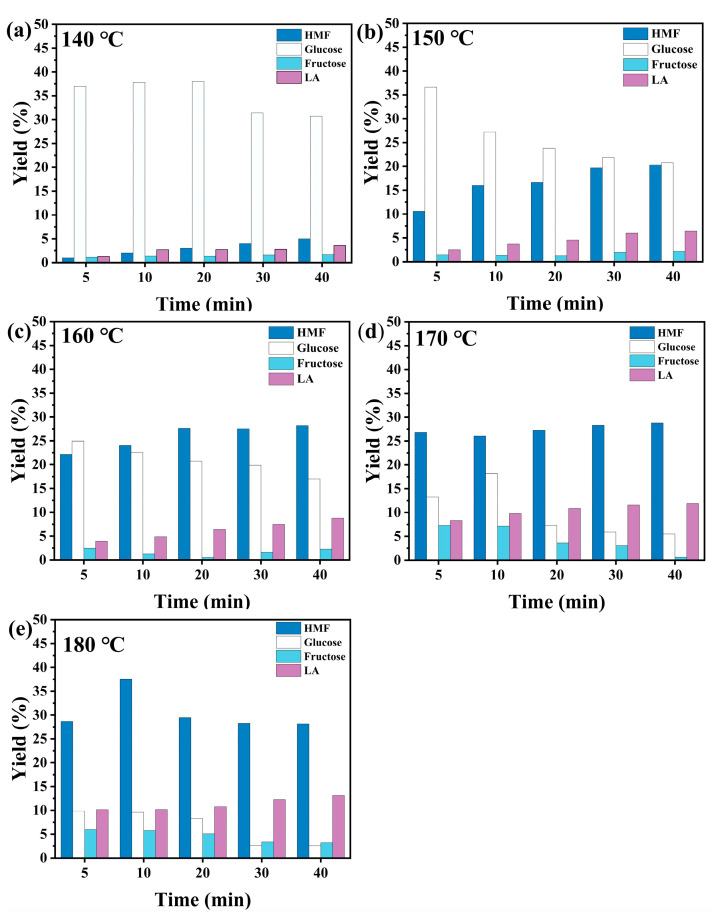
HMF, glucose, fructose, and LA yields from starch catalyzed by P_0.5_/BM_1.0_ in different reaction times and temperatures. (Condition: 0.15 g starch, P_0.5_/BM_1.0_ concentration of 0.225%, 8 mL CC/FA, 12 mL EAC).

The reaction time and reaction temperature are two of the most important influencing factors in determining the yield of catalytic starch conversion to HMF. Therefore, the effects of the reaction temperature and time on the conversion of catalytic starch to HMF were investigated. As can be seen in Figure 6, the reaction temperature and time had a significant influence on the HMF, glucose, fructose, and LA yields. When the reaction was carried out at 140 °C for 5 min, the yields of HMF, glucose, fructose, and LA were 3.23%, 36.97%, 1.16%, and 1.30%, respectively. When the temperature was increased to 180 °C, the yields of HMF, glucose, fructose, and LA were 28.67%, 9.84%, 5.95%, and 10.15%, respectively. In our previous work, the maximum yield of HMF in the catalytic system of P_0.5_/BM_1.0_/DESs/EAC increased with the reaction temperature and the yield of glucose decreased. Moreover, the yield of HMF increased over time when the temperature was below 160 °C, but then decreased when the temperature exceeded 170 °C. The highest HMF yield of 37.94% was obtained when the reaction time was 10 min. These results suggest that heterogeneous catalysts need higher reaction temperatures to achieve their maximum catalytic activity.

### 2.5. Preparation of HMF by Catalytic Conversion of Glucose

**Figure 7:**
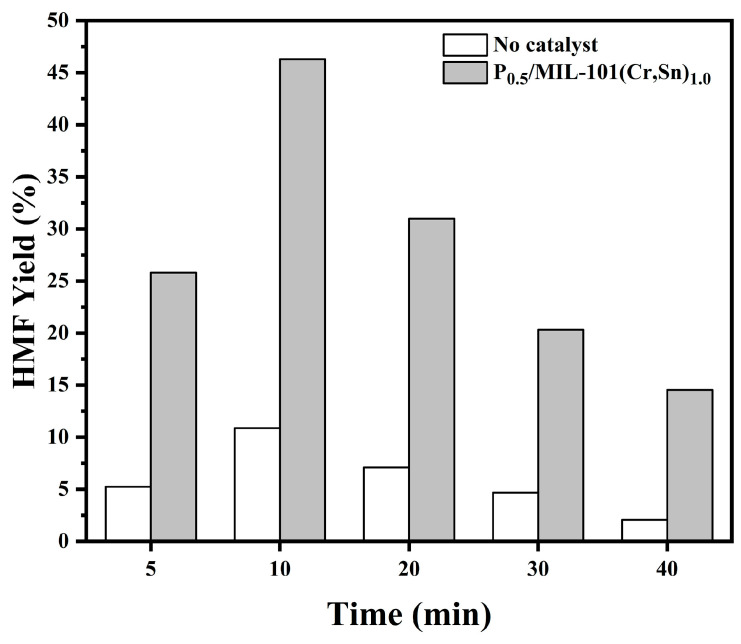
Preparation of HMF by glucose catalytic conversion.

In a previous study [28], we used Lewis acidic bimetallic MOFs with Brønsted acid H_2_SO_4_ as catalysts for the conversion of glucose and starch to HMF, in a mixture of saturated sodium chloride solution and GVL. We found that bimetallic MOFs are essential in the catalytic reaction to isomerize glucose to fructose. The bimetallic MOFs and H_2_SO_4_ in combination had a synergistic effect on the increase in the HMF yield. Therefore, the P_0.5_/BM_1.0_ material with Brønsted–Lewis biphasic acidity was used as a catalyst to convert glucose in a biphasic system consisting of 8 mL of CC/FA (1:2) DESs and 12 mL of EAC. This was achieved with microwave heating at 140 °C for different reaction times (Figure 7). The yield of HMF prepared with glucose as the substrate was lower when no catalyst was used. The highest yield of HMF was only 10.78% when the reaction proceeded for 10 min. Under the same conditions, the highest yield of HMF was achieved at 46.29% with the addition of the P_0.5_/BM_1.0_ catalyst. The total acid amount in the P_0.5_/BM_1.0_ catalyst was 6.15 μmol/g, as determined by pyridine infrared spectroscopy. The Brønsted acid content was 29% of the Lewis acid amount (Table 1), demonstrating once again the synergistic effect of Brønsted and Lewis acids in combination when preparing HMF from glucose-based substrates. In a previous work, using H_2_SO_4_ and BM_0.1_ as cocatalysts, the yield of HMF was 60.75% when reacting with glucose at 140 °C for 60 min, which was much higher than that using P_0.5_/BM_1.0_ as the catalyst (46.29%). One possible reason is that H_2_SO_4_ is a liquid acid that can make contact with the reactants more fully in a catalytic reaction, leading to an increased yield of HMF. Furthermore, the viscosity of a DES-based reaction system is larger compared with that of the organic solvent–brine system, which affects the mass transfer, and thus, the yield of HMF is lower.

### 2.6. Recycling of Catalyst

**Figure 8:**
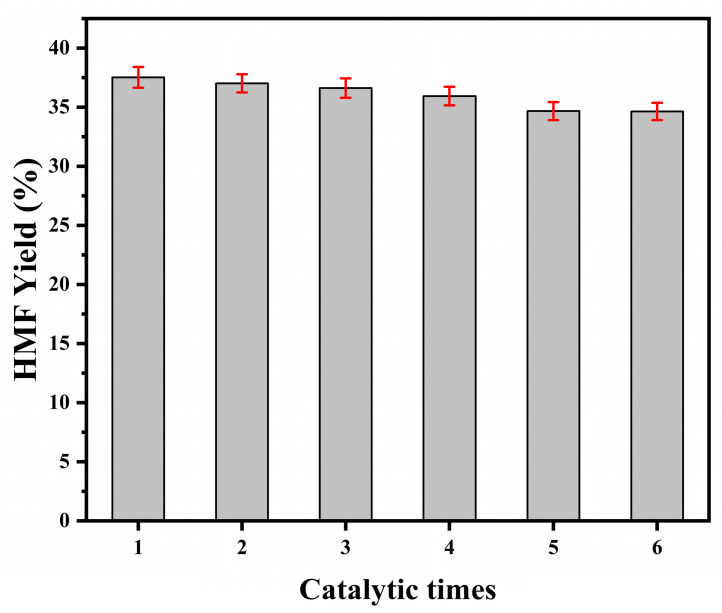
P_0.5_/BM_1.0_ reusability for starch conversion. (Condition: P_0.5_/BM_1.0_ concentration of 0.225%,180 °C, 10 min, 8 mL CC/FA, 12 mL EAC).

The recyclability of heterogeneous catalysts is a major advantage over working with homogeneous catalysts. To evaluate the recyclability of the P_0.5/_BM_1.0_ catalyst, the reaction was carried out six times at 180 °C for 10 min. In our work, P_0.5_/BM_1.0_ was separated from the reaction mixture by centrifugation, washed two to three times with ethanol and deionized water, then dried in a vacuum oven at 60 °C for 2 h. Subsequently, the recovered P_0.5_/BM_1.0_ was added into a fresh reaction solution (8 mL CC/FA and 12 mL EAC) for the next reaction. As shown in Figure 8, P_0.5_/MIL-101(Cr, Sn)_1.0_ retained good catalytic activity for the conversion of starch to HMF with a yield of 35.18% after six cycles. The slight decrease (2.88%) in the HMF yield was possibly due to the domain-limiting effect of the MOF material [50] and the interaction between the PTA and the carrier, making it difficult for the PTA present inside the skeleton to be shed from the pore cage of the MOFs.

### 2.7. Chemical Pathway of Starch Conversion to HMF

**Scheme 1:**
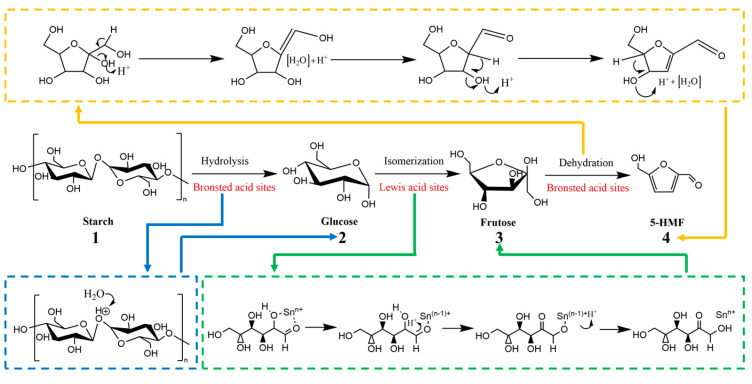
Proposed reaction pathway for the conversion of starch in the catalytic system.

Based on the properties and experimental studies of P_0.5_/BM_1.0_, a possible catalytic mechanism is proposed [51], which is mainly divided into three steps, as shown in Scheme 1: first, starch molecules are hydrolyzed under acidic conditions, and glucoside bonds are destroyed to produce glucose; second, glucose is isomerized to fructose; finally, the fructose molecule removes three water molecules to form HMF. In the second process, Sn ^n+^ electrophiles attack the glucose molecule, acting on the oxygen atoms of the aldehyde group to form an intermediate, which is then isomerized to fructose. At the Brønsted acid site, the hydroxyl group of the fructose molecule and three water molecules are removed to form HMF.

## 3. Materials and Methods

### 3.1. Materials

Tapioca starch was sourced from Guangxi Hong Feng Starch Co., Ltd. (Nanning, China) and was dried to constant weight and stored in a desiccator prior to use. Tin chloride (SnCl_4_), chromium (III) nitrate nonahydrate (Cr(NO_3_)_3_·9H_2_O), *p*-phthalic acid, and phosphotungstic acid (PTA) were purchased from Aladdin (all AR grade). 5-Hydroxymethyl-2-furaldehyde, methanol, and acetate were purchased from Shanghai Aladdin Biochemical Technology Co., Ltd. (Shanghai, China), all of which were of HPLC grade.

### 3.2. Preparation of DESs

A series of deep eutectic solvents were prepared by mixing components in certain molar ratios and stirring at 100 °C [52]. The DESs used in this study were choline chloride/formic acid (CC/FA), choline chloride/lactic acid (CC/LA), choline chloride/glycerol (CC/Gly), ethylamine hydrochloride/glycol (ET/EC), and choline chloride/diethanolamine (CC/DEA). Table S1 details the molar ratios of the DES components.

### 3.3. Catalyst Preparation

MIL-101(Cr, Sn)_1.0_ (BMOFs) were synthesized according to our previous work [28]. Typically, Cr(NO_3_)_3_·9H_2_O (7 mmol), SnCl_4_ (1 mmol), *p*-phthalic acid (8 mmol), acetate (3 mL) were dissolved in 48 mL H_2_O and dispersed using ultrasound. PTA/MIL-101(Cr, Sn)_1.0_ was synthesized in part with reference to the literature [27]. PTA was dried at 130 °C for 4 h before use. Then, 0, 0.5, 1.0, 2.0, or 3.0 g PTA was added to the above solution. The mixture was transferred to a 100 mL PPL-lined hydrothermal reactor and heated at 200 °C for 12 h. After the reaction was cooled down to room temperature, the solid products were separated by centrifugation, washed with water, ethanol, then washed with DMF, and dried at 60 °C for 6 h. As an additional purification step, the product was centrifuged again, washed with deionized water, and dried at 60 °C for 6 h under vacuum. Depending on the amount of PTA introduced into the synthesis mixture, PTA/MIL-101(Cr, Sn)_1.0_ with different PTA loadings were obtained and labeled as P_Y_/MIL-101(Cr, Sn)_1.0_ (Y = 0, 0.5, 1.0, 2.0, 3.0), abbreviated as P_Y_/BM_1.0_.

### 3.4. Characterization

Elemental analysis was performed using an atomic absorption spectrometer (contrAA 800 D, (Analytik Jena, Jena, Germany). The test conditions were flame mode, acetylene–air flame type, and combustion head: 100 mm. The BET surface area, pore size, and volume of the samples were measured using a specific surface and pore size analyzer (ASAP-2460 Micromeritics, Norcross, GA, USA) with a multipoint method on the basis of nitrogen adsorption–desorption isotherms at a degassing temperature of 150 °C, with degassing for 12 h. The sample surface composition and chemical state were analyzed using a K-Alpha X-ray photoelectron spectrometer (Thermo Fisher Scientific, Waltham, MA, USA). The test parameters were Al Kα-rays (*hv* = 1486.6 eV), operating voltage: 12 kV, filament current: 6 mA, narrow spectrum with signal accumulation of 5 or more cycles, and charge correction with C1s = 284.80 eV binding energy as energy standard. Powder crystal structure: Mini Flex 600 (MINIFLEX 600 Rigaku, Tokyo, Japan). The test conditions were voltage: 40 kV, test current: 40 mA, scan speed 10°/min, scan range: 5–80°. Brønsted and Lewis acid analysis: Nicolet iS50 pyridine infrared analyzer (Thermo Fisher Scientific, Waltham, MA, USA).

Samples were analyzed for Brønsted acid and Lewis acid content using a Nicolet iS50 pyridine infrared analyzer (Thermo Fisher Scientific, Waltham, MA, USA) [53,54]. The thermal stability of the catalyst was measured using an STA 2500 thermogravimetric analyzer (NETZSCH, Selb, Germany). The sample was heated in an air atmosphere from room temperature to 600 °C at a rate of 10 °C/min.

### 3.5. Acid Strength and Density of Catalysts

P_Y_/BM_1.0_ (0.10 g) was added to 30 mL of anhydrous acetonitrile solution and stirred at room temperature for 12 h. The pH of the supernatant liquid was measured using a precision pH meter, and the initial potentials of various acids were in the following ranges: (1) weak acids: −100 to 0 mV; (2) strong acids: 0 to 100 mV; (3) ultra-strong acids: >100 mV [24].

The acid density of the sample was determined by acid–base titration. P_Y_/BM_1.0_ (0.10 g) was added to 20 mL of NaCl solution (20 mmol/L) and stirred at room temperature for 24 h. The supernatant was titrated with 5 g/L phenolphthalein indicator solution and 5 mmol/L NaOH solution [50]. The acid density was calculated using the following equation (Equation (1)):

(1) $$\mathrm{Acid}\;\mathrm{density}=\frac{C\times V}{1000\;m}$$

where:

*C* is concentration of NaOH solution (mmol/L);

*V* is volume of NaOH consumed (mL);

*m* is mass of the added sample (g).

### 3.6. Dehydration of Starch to HMF

The catalytic experiments were carried out in a 100 mL PEEK microwave deca-parallel high-pressure synthesis reactor with PTFE liner at a microwave frequency of 2450 MHz. Typically, starch, the P_Y_/BM_1.0_ catalyst, and 20 mL each of the DESs and ethyl acetate (EAC) were added to the reaction vessel. The mixture was stirred continuously for 10 min at a constant speed of 100 r/min, at a given temperature of 140 °C to 180 °C, and a microwave output power of 800 W. After the reaction was complete, the air-cooled automatic cooling program was initiated. When the pressure in the tank was at atmospheric pressure, the mixture was removed and centrifuged, with the supernatant solution being retained for analysis. Each experiment was repeated three times, with an average value being quoted for experimental results.

HMF was analyzed by high-performance liquid chromatography (HPLC) (Agilent 1260, Agilent Technologies, Santa Clara, CA, USA) with an SB-C18 column (4.6 × 250 mm, Agilent Technologies, Santa Clara, CA, USA) and a UV detector. One hundred microliters of the reaction solution was diluted to 10 mL with deionized water, passed through a 0.22 μm organic filter membrane, then placed in a 1.5 mL injection vial for sampling. The mobile phase was 1% glacial acetic acid solution (*v/v*) and methanol, at a volume ratio of 9:1 [54]. The glucose, fructose, and levulinic acid (LA) concentrations were also determined by HPLC with a BIO-RAD Aminex HPX-87H (7.8 mm × 300 mm; Bio-Rad Laboratories, Hercules, CA, USA) column and differential refractive index detector. The mobile phase was 5 mM H_2_SO_4_ solution at a flow rate of 0.6 mL/min, a column temperature of 50 °C, and a refractive index detector temperature of 40 °C, with a run time of 30 min [55].

The yields of HMF, glucose, fructose, and LA were calculated using the following equations (Equation (2)–(5)):

HMF yield (mol%):

(2) $${\mathrm{HMF}}_{\mathrm{yield}}=\frac{\mathrm{moles}\;\mathrm{of}\;\mathrm{HMF}\;\mathrm{in}\;\mathrm{solution}}{\mathrm{moles}\;\mathrm{of}\;\mathrm{starch}\;\mathrm{in}\;\mathrm{the}\;\mathrm{reaction}}\times 100\%$$

Yields of glucose, fructose, and LA (wt%):

(3) $${\mathrm{Glucose}}_{\mathrm{yield}}=\frac{\mathrm{mass}\;\mathrm{of}\;\mathrm{remaining}\;\mathrm{glucose}}{\mathrm{mass}\;\mathrm{of}\;\mathrm{starting}\;\mathrm{starch}}\times 100\%$$

(4) $${\mathrm{Fructose}}_{\mathrm{yield}}=\frac{\mathrm{mass}\;\mathrm{of}\;\mathrm{remaining}\;\mathrm{fructose}}{\mathrm{mass}\;\mathrm{of}\;\mathrm{starting}\;\mathrm{starch}}\times 100\%$$

(5) $${\mathrm{LA}}_{\mathrm{yield}}=\frac{\mathrm{mass}\;\mathrm{of}\;\mathrm{remaining}\;\mathrm{LA}}{\mathrm{mass}\;\mathrm{of}\;\mathrm{starting}\;\mathrm{starch}}\times 100\%$$

## 4. Conclusions

In this study, P_Y_/BM_1.0_ catalysts were prepared via the in situ synthesis of PTA-encapsulated, highly stable Sn–Cr bimetallic MOFs. The conversion of starch to HMF was investigated in a DES/EAC mixed solvent and found to be efficient; furthermore, a moderate HMF yield of 37.94% was obtained from starch in the presence of P_0.5_/MIL-101(Cr, Sn)_1.0_. The catalyst was stable and had adequate recyclability. After six cycles, the HMF yield decreased by only 2.88%. Employing a DES as a cocatalyst and solvent reduced the use of organic solvents. P_Y_/BM_1.0_ and DES are an adequate combination for the conversion of starch to HMF. The one-pot synthesis proposed herein has potential applications in the large-scale production of HMF from carbohydrates.

## Figures and Tables

**Table 1:** Acid strength, acid density, and B/L acid ratio of samples.

Catalysts	Initial Potential	Acid Density	Acid Density and B/L Acid Ratio ^a^		Surface Atomic Content ^b^			PTA Loading ^c^
	(mV)	(mmol/g)	(μmol/g)	B/L	ω (O)%	ω (P)%	ω (W)%	(wt%)
PTA	588	6.47	20.92	2.82	22.22	1.08	76.67	100
BM_1.0_	−45	0.03	5.15	0.02	33.57	N/A	N/A	0
P_0.5_/BM_1.0_	142	1.16	6.15	0.29	35.97	0.45	1.55	9.71
P_1.0_/BM_1.0_	180	1.82	6.45	0.48	35.41	0.56	2.04	12.78
P_2.0_/BM_1.0_	195	2.12	7.20	0.93	39.98	1.04	3.28	20.59
P_3.0_/BM_1.0_	193	1.98	6.82	0.73	38.80	0.90	3.03	18.98

**^a^** The acid density and the ratio of Brønsted and Lewis acids of the samples were measured by pyridine infrared spectroscopy. **^b^** The elemental content of the sample surface was measured by XPS. **^c^** The loading of PTA was calculated based on the content of W atoms.

**Table 2:** Analysis of specific surface area, pore size, and pore volume of samples.

Samples	*S*_BET_ ^a^	*V*_total_ ^b^	Pore Size ^c^
	(m^2^/g)	(cm^3^/g)	(nm)
BM_1.0_	2826	1.31	4.02
P_0.5_/BM_1.0_	1379	0.84	3.41
P_1.0_/BM_1.0_	1171	0.74	3.97
P_2.0_/BM_1.0_	1134	0.62	2.66
P_3.0_/BM_1.0_	895	0.48	2.58

**^a^** Specific surface area obtained by BET specific surface area test method. **^b^** Hole volume of the sample. **^c^** Average pore size of the sample.

**Table 3:** Effect of the volume ratio of DESs to EAC on the yield and separation coefficient of HMF.

DESs/EAC (*v/v*)	HMF in EAC (mg)	HMF in DES (mg)	HMF Yield (%) ^a^	Partition Coefficient (%) ^b^
2/18	17.69	4.75	21.94	76.78
4/16	16.86	11.56	27.09	59.25
6/14	18.54	19.45	36.18	48.81
8/12	14.92	24.91	37.94	37.46
10/10	14.46	22.26	34.97	39.38

^a^ Reaction conditions: 0.15 g starch, P_0.5_/BM_1.0_ concentration of 0.225%, 20 mL of reaction solution, 180 °C, 10 min. ^b^ Partition coefficient = HMF in EAC/total weight of HMF.

## Data Availability

The data supporting the findings of this study are available from the corresponding author upon request.

## Supplementary Materials

The following supporting information can be downloaded at: https://www.mdpi.com/article/10.3390/ijms241411480/s1.
