# Supplementary material for: Catalytic Conversion of Carbohydrates into 5-Hydroxymethylfurfural by Phosphotungstic Acid Encapsulated in MIL-101 (Cr, Sn) Catalyst in Deep Eutectic Solvents
Source: Int J Mol Sci. 2023 Jul 14;24(14):11480. doi: 10.3390/ijms241411480 (PMC10380470; doi:10.3390/ijms241411480)
Supplement: Supplementary file 1 [file ijms-24-11480-s001.zip › ijms-2498718-supplementary.pdf]

Catalytic conversion of carbohydrates into 5-hydroxymethyl-furfural by phosphotungstic acid encapsulated in MIL-101 (Cr, Sn) catalyst in deep eutectic solvents

Wei Mao † [1], Jiawen Hao † [1], Lingyu Zeng [1], Hao Wang [1], Hao Xu [1], Jinghong Zhou [1,*]

[1] Guangxi Key Laboratory of Clean Pulp & Papermaking and Pollution Control, School of Light Industrial and Food Engineering, Guangxi University, Nanning 530004, China.

† These authors contribute equally to this work

* Correspondence: jhzhoudou@gxu.edu.cn

Table S1 Viscosity of DESs and its solubility to starch

DESs	HBA [a]	HBD [b]	Abbreviation	mole ratio	Viscosity (m Pa.s)	Starch dissolution [c] (g $_{DES}$/g $_{starch}$)
Acidic	Choline chloride	formic acid	CC/FA	1:2	11.31	0.7085
	Choline chloride	lactic acid	CC/LA	1:2	53.13	0.1036
Neutral	Choline chloride	glycerol	CC/Gly	1:2	38.60	0.1281
	Ethylamine hydrochloride	glycol	ET/EC	1:2	51.50	0.0638
Alkalinity	Choline chloride	Diethanolamine	CC/DEA	1:8	106.70	0.1134

[a] HBA: Hydrogen bond acceptor. [b] HBD: Hydrogen bond donor. [c] Tapioca starch was added to the continuous DESs at a temperature of 100 °C for 2 h until it could not be dissolved. Observe with a polarized light microscope, and if a central cross appears, no more starch is added.

Table S2 Solubility determination of DESs and extractants

Entry	Organic solvent	V_1 (mL)	V_2 (mL)	DES exist or not
1	MIBK	5	5	-
2	Ethanol	5	/	+
3	Ethyl acetate	5	5	-
4	Acetonitrile	5	/	+
5	Isopropanol	5	/	+
6	γ-Valerolactone	5	/	+

V_1: volume of DES added; V_2: volume of DES after strong stirring and separation of DES and extractant; /: indicates that DES and extractant are miscible without separation; +: indicates that there is DES residue after evaporation of organic extractant; -: indicates that there is no DES residue after evaporation of organic extractant.
